# Multi‐Trajectories of Sleep Disturbance and Depressive Symptoms and Incident Osteoarthritis: Evidence From the English Longitudinal Study of Ageing

**DOI:** 10.1002/brb3.71788

**Published:** 2026-09-23

**Authors:** Shufang Deng, Zeping Chen, Wei Zhao, Zhaoheng Chen, Guimin Zhang, Peijuan Li, Ningyuan Lai, Rui Xie

**Affiliations:** ^1^ Department of Tuina No.3 Affiliated Hospital of Chengdu University of Traditional Chinese Medicine (West District), Chengdu Pidu District Hospital of Traditional Chinese Medicine Chengdu China

**Keywords:** osteoarthritis, sleep disturbance, depressive symptoms, group‐based multi‐trajectory modelling, English Longitudinal Study of Ageing

## Abstract

**Background:**

Osteoarthritis (OA) is a leading cause of pain and disability in older adults. Sleep disturbance and depressive symptoms are common and have been linked to multiple chronic conditions, yet how their co‐developing longitudinal patterns relate to OA onset remains unclear. We aimed to identify joint trajectories of sleep disturbance and depressive symptoms and examine their associations with incident OA.

**Methods:**

Data were drawn from the English Longitudinal Study of Ageing (ELSA), a nationally representative prospective cohort of community‐dwelling adults aged ≥ 50 years. Sleep disturbance and depressive symptoms (CES‐D) assessed across three waves over eight years were modeled using group‐based multi‐trajectory modeling (GBMTM). OA incidence was defined as new‐onset OA between Wave 8 and Wave 9 among participants free of OA at Wave 8. Multivariable logistic regression was used to estimate odds ratios (ORs) and 95% confidence intervals (CIs), adjusting for sociodemographic factors, health behaviors, body mass index, and comorbidities.

**Results:**

A total of 3604 participants were included. Four joint trajectory groups were identified: persistently good sleep/no depressive symptoms; persistently moderate sleep disturbance/low depressive symptoms; persistently poor sleep/moderate depressive symptoms; and persistently poor sleep/persistently high depressive symptoms. Compared with the persistently good sleep/no depressive symptoms group, the fully adjusted odds of incident OA were higher in the persistently poor sleep/persistently high depressive symptoms group (OR = 2.85, 95% CI 1.90–4.28), the persistently poor sleep/moderate depressive symptoms group (OR = 2.14, 95% CI 1.52–3.02), and the persistently moderate sleep disturbance/low depressive symptoms group (OR = 1.56, 95% CI 1.20–2.03).

**Conclusion:**

Long‐term co‐occurrence of sleep disturbance and depressive symptoms was associated with incident OA in middle‐aged and older adults. These patterns may help identify adults at elevated risk, but causal effects and the preventive value of sleep or depression interventions require confirmation.

## Graphical abstract

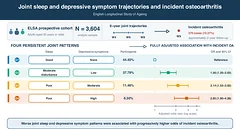

## 1 Introduction

Osteoarthritis (OA) is a leading global cause of pain and disability and a major contributor to chronic pain and functional limitation in middle‐aged and older adults (Hunter and Bierma‐Zeinstra 2019; Kloppenburg et al. 2025). Recent analyses from the global burden of disease (GBD) study indicate that the number of people living with OA has continued to increase over the past three decades, with disability burden rising in many countries, largely driven by population ageing (Wong et al. 2023; Xie et al. 2025). Because OA incidence and disability accelerate after age 50, its public health impact extends well beyond local joint symptoms, contributing substantially to loss of healthy life expectancy and healthcare utilization (Wong et al. 2023).

Traditionally, OA prevention and management have focused on structural joint degeneration and its mechanical consequences. Increasing evidence, however, suggests that OA onset and progression reflect a broader interplay of multi‐system and modifiable factors rather than a purely “joint‐centered” process (Hunter et al. 2020). Sleep disturbance and depressive symptoms are among the most common mental health problems in later life (Canever et al. 2024; Hu et al. 2022). Both are linked to inflammatory activity, pain processing, weight regulation, and health behaviors—pathways that are biologically and behaviorally relevant to OA—making them plausible upstream targets for prevention (Figorilli et al. 2025; Irwin 2025; Zou et al. 2024). Longitudinal studies have shown that poor sleep quality and atypical sleep duration are associated with a higher risk of incident OA (Wang et al. 2025). Genetic evidence has further suggested potential causal effects of insomnia and short sleep on OA risk (Ni et al. 2022), strengthening the rationale for viewing sleep as an actionable upstream factor.

Depressive symptoms are also closely related to OA. Clinical and population‐based studies consistently associate depression with greater OA pain, functional limitation, and disease burden, potentially through pain sensitization, reduced physical activity, and poorer treatment adherence (Wang and Ni 2022). Genetic studies have also examined depression as an exposure in its own right. A bidirectional Mendelian randomization analysis found that genetic liability to major depressive disorder was associated with greater OA risk and identified shared genetic liability between the two conditions (Zhang et al. 2022). A subsequent two‐step Mendelian randomization study reported a similar direction of association, with whole‐body fat mass accounting for part of the estimated effect (Yan et al. 2024). These findings support a possible contribution of depression to OA development, although causal interpretation remains dependent on Mendelian randomization assumptions. Sleep disturbance and depression frequently co‐occur and may reinforce each other over time (Li et al. 2022). Sleep problems can increase subsequent depression risk, while depressive symptoms may worsen sleep and undermine health behaviors. Recent ELSA work suggests that depressive symptoms may mediate part of the sleep–OA association (Wang et al. 2025).

Despite this, much of the existing evidence has examined sleep or depression in isolation, or relied on cross‐sectional associations, which cannot capture how these conditions co‐evolve across later life or how their joint patterns shape longer term OA risk. Sleep disturbance and depressive symptoms may cluster into distinct joint trajectories, potentially producing cumulative risk, yet evidence linking such multi‐trajectory patterns to incident OA remains limited (Wang et al. 2025). Therefore, using the English Longitudinal Study of Ageing (ELSA) (Steptoe et al. 2012), we applied group‐based multi‐trajectory modeling (GBMTM) to identify joint trajectories of sleep disturbance and depressive symptoms across an eight‐year period, and examined their associations with subsequent incident OA (Nagin et al. 2024). By characterizing modifiable sleep–mental health patterns, this study aims to inform early risk stratification and integrated prevention strategies for OA in aging populations.

## 2 Methods

### 2.1 Study Design and Population

This population‐based prospective cohort study used data from the ELSA, an ongoing nationally representative study of adults aged ≥ 50 years in England (Steptoe et al. 2012). ELSA began in 2002 (wave 1), with follow‐up approximately every two years. At Wave 1, ELSA completed 12,099 interviews, including 11,391 core sample members (Steptoe et al. 2012). Completed interviews at Waves 2–9 numbered 9432, 9771, 11,050, 10,274, 10,601, 9666, 8445, and 8736, respectively. These totals do not represent simple attrition because refreshment samples were introduced at later waves. The study received ethical approval from the National Research Ethics Service, and all participants provided written informed consent.

**FIGURE 1:**
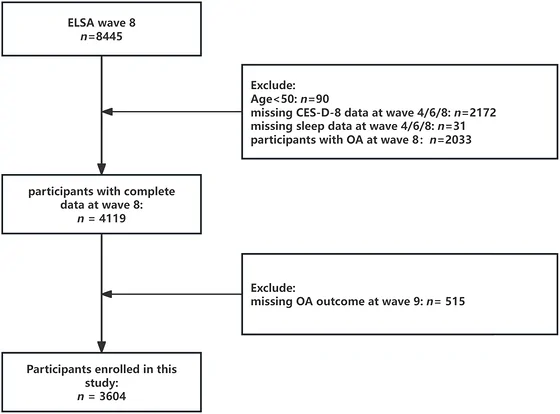
Research flowchart.

We drew on data from Wave 4 (2008–2009) to Wave 9 (2018–2019). Because sleep‐related items were assessed more systematically from Wave 4 onward, sleep and depressive symptom measures from Waves 4, 6 (2012–2013), and 8 (2016–2017) were used to derive 8‐year joint trajectories. For risk estimation, Wave 8 was defined as the index (baseline) wave, and incident OA was ascertained at Wave 9. Participants were eligible if they: (1) were aged ≥50 years; (2) had sleep and depressive symptom data at Waves 4, 6, and 8; (3) reported no OA at Wave 8; and (4) had OA outcome information at Wave 9. The final analytic sample included 3604 participants (Figure 1).

### 2.2 Assessment of Exposures

#### 2.2.1 Sleep Disturbance

Sleep disturbance was assessed using four questionnaire items capturing overall sleep quality and insomnia‐related symptoms: overall sleep satisfaction, difficulty initiating sleep, difficulty returning to sleep after nocturnal awakening, and waking feeling tired. Each item was rated on a Likert scale; item scores were summed to create a sleep disturbance score ranging from 4 to 16, with higher scores indicating worse sleep (Jenkins et al. 1988; Wang et al. 2025; Yu et al. 2025). These data from Waves 4, 6, and 8 were used to model longitudinal sleep trajectories.

#### 2.2.2 Depressive Symptoms

Depressive symptoms were measured using the 8‐item Center for Epidemiologic Studies Depression Scale (CES‐D‐8) (Missinne et al. 2014). Participants reported the presence of eight symptoms over the past week; scores ranged from 0 to 8, with higher scores indicating more severe depressive symptoms. CES‐D‐8 data from Waves 4, 6, and 8 were used to model depressive symptom trajectories.

### 2.3 Outcome

The primary outcome was incident OA between Wave 8 and Wave 9 based on self‐reported doctor‐diagnosed OA. Incident OA was defined as no OA at Wave 8 and the first report of OA at Wave 9.

### 2.4 Covariates

Potential confounders were measured at the index wave (Wave 8), including:

#### 2.4.1 Sociodemographics

Age (continuous), gender (male/female), ethnicity (White/non‐White), and marital status (married/other).

#### 2.4.2 Socioeconomic Status

Education (low/middle/high), household wealth (total income in quintiles).

#### 2.4.3 Health Behaviors

Physical activity (low/high), smoking (yes/no), and alcohol consumption (≥1 time/week vs <1 time/week).

#### 2.4.4 Health Status

Body mass index (BMI, kg/m^2^; continuous) and comorbidities (yes/no; e.g., hypertension, diabetes, and chronic respiratory disease).

To maintain the integrity of the data, missing data were imputed via multiple imputation. The missing data proportions are shown in Table S1.

### 2.5 Statistical Analysis

#### 2.5.1 Multi‐Trajectories Modeling

**FIGURE 2:**
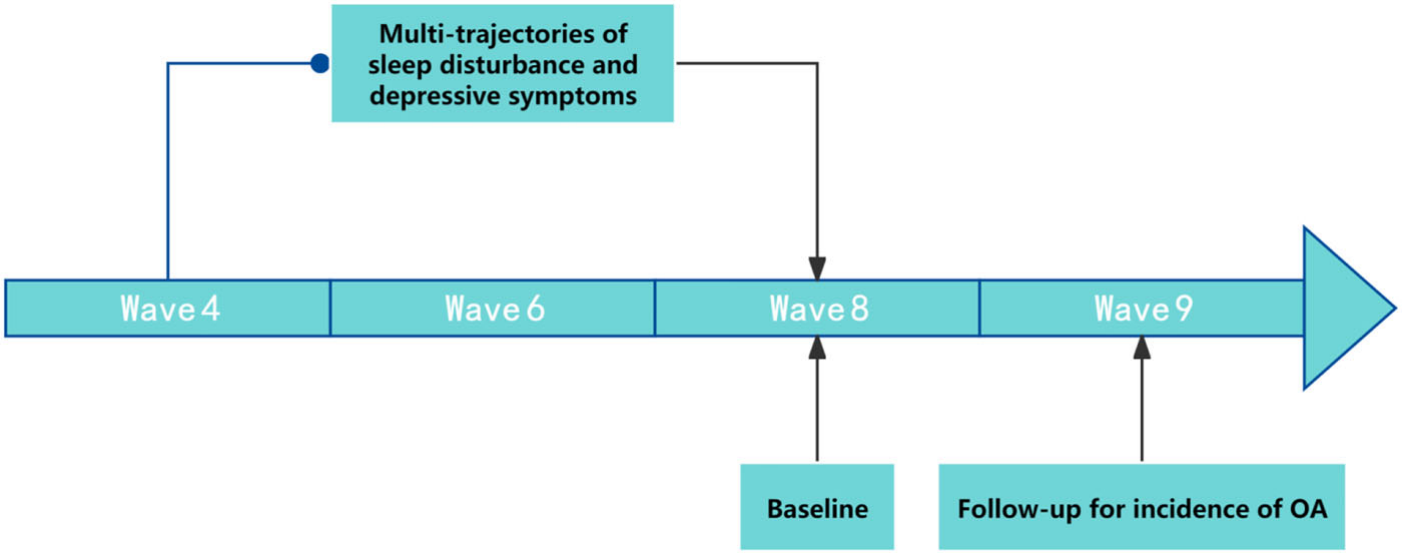
Time frame for the analyses of multi‐trajectories of sleep disturbance and depressive symptoms findings.

We applied group‐based multi‐trajectory modeling (GBMTM) to jointly model sleep disturbance scores and CES‐D‐8 scores across Waves 4, 6, and 8, identifying latent subgroups with distinct joint trajectories over eight years (Figure 2). Models with 2 to 4 groups were compared, and the optimal solution was selected based on the Bayesian information criterion (BIC), average posterior probability (> 0.70), minimum group size (> 5%), and clinical interpretability. Four joint trajectory groups were retained: persistently good sleep/no depressive symptoms; persistently moderate sleep disturbance/low depressive symptoms; persistently poor sleep/moderate depressive symptoms; and persistently poor sleep/persistently high depressive symptoms.

#### 2.5.2 Association With incident OA

Baseline characteristics were summarized using means (SD), medians (IQR), or frequencies (%), as appropriate. Group differences were evaluated using χ^2^ tests for categorical variables, one‐way ANOVA for normally distributed continuous variables, or Kruskal–Wallis tests for non‐normal continuous variables.

To examine the association between joint trajectory group membership and incident OA from Wave 8 to Wave 9, we fitted binary logistic regression models and reported odds ratios (ORs) with 95% confidence intervals (CIs). Three models were built sequentially: Model 1 unadjusted (crude) model including only the exposure; Model 2 additionally adjusted for demographic factors; and Model 3 further adjusted for lifestyle as well as health variables.

Analyses were performed in R software (version 4.3.0) and SAS 9.4 software. All tests were two‐sided, and *p* < 0.05 was considered statistically significant.

Subgroup analyses were exploratory and examined age (< 65/≥ 65 years), gender, marital status, education, household wealth, smoking, alcohol consumption, physical activity, and BMI (< 30/≥ 30 kg/m^2^). Differences across strata were evaluated using *p* values for interaction.

## 3 Results

### 3.1 Baseline Characteristics

A total of 3604 eligible participants were included (Table 1). At the index wave (Wave 8), the mean age was 69.99 years (SD 7.95), and 51.94% were women. Most participants were White (96.73%). The mean BMI was 27.67 kg/m^2^ (SD 5.14). Over approximately 2 years of follow‐up from Wave 8 to Wave 9, 370 participants (10.27%) developed incident OA.

**TABLE 1:** Baseline characteristics of study populations across multi‐trajectories of sleep disturbance and depressive symptoms.

Variable	Levels	Overall	Group 1	Group 2	Group 3	Group 4	*p*‐value
		*n *= 3604	*n* = 1602	*n *= 1362	*n *= 413	*n* = 227	
Age, mean (sd)		69.99 (7.95)	70.17 (7.92)	70.22 (7.96)	69.18 (8.02)	68.91 (7.81)	0.008
BMI, mean (sd)		27.67 (5.14)	27.41 (4.87)	27.64 (5.02)	28.24 (5.50)	28.72 (6.69)	0.015
Race, *n* (*p*%)							< 0.001
	Non‐white	118.00 (3.27%)	55.00 (3.43%)	35.00 (2.57%)	10.00 (2.42%)	18.00 (7.93%)	
	White	3486.00 (96.73%)	1547.00 (96.57%)	1327.00 (97.43%)	403.00 (97.58%)	209.00 (92.07%)	
Gender, *n* (*p*%)							< 0.001
	Female	1872.00 (51.94%)	696.00 (43.45%)	742.00 (54.48%)	281.00 (68.04%)	153.00 (67.40%)	
	Male	1732.00 (48.06%)	906.00 (56.55%)	620.00 (45.52%)	132.00 (31.96%)	74.00 (32.60%)	
Marital, *n* (*p*%)							< 0.001
	Married	2424.00 (67.26%)	1,135.00 (70.85%)	928.00 (68.14%)	258.00 (62.47%)	103.00 (45.37%)	
	Other	1180.00 (32.74%)	467.00 (29.15%)	434.00 (31.86%)	155.00 (37.53%)	124.00 (54.63%)	
Education, *n* (*p*%)							< 0.001
	Below high school	1053.00 (29.22%)	412.00 (25.72%)	400.00 (29.37%)	134.00 (32.45%)	107.00 (47.14%)	
	College or above	1709.00 (47.42%)	822.00 (51.31%)	659.00 (48.38%)	163.00 (39.47%)	65.00 (28.63%)	
	High school	842.00 (23.36%)	368.00 (22.97%)	303.00 (22.25%)	116.00 (28.09%)	55.00 (24.23%)	
Income, *n* (*p*%)							< 0.001
	Q1	724.00 (20.09%)	290.00 (18.10%)	257.00 (18.87%)	100.00 (24.21%)	77.00 (33.92%)	
	Q2	719.00 (19.95%)	300.00 (18.73%)	272.00 (19.97%)	96.00 (23.24%)	51.00 (22.47%)	
	Q3	719.00 (19.95%)	292.00 (18.23%)	286.00 (21.00%)	89.00 (21.55%)	52.00 (22.91%)	
	Q4	721.00 (20.01%)	331.00 (20.66%)	286.00 (21.00%)	74.00 (17.92%)	30.00 (13.22%)	
	Q5	721.00 (20.01%)	389.00 (24.28%)	261.00 (19.16%)	54.00 (13.08%)	17.00 (7.49%)	
Smoke, *n* (*p*%)							< 0.001
	No	3277.00 (90.93%)	1474.00 (92.01%)	1252.00 (91.92%)	369.00 (89.35%)	182.00 (80.18%)	
	Yes	327.00 (9.07%)	128.00 (7.99%)	110.00 (8.08%)	44.00 (10.65%)	45.00 (19.82%)	
Drink, *n* (*p*%)							< 0.001
	<1/week	1512.00 (41.95%)	601.00 (37.52%)	566.00 (41.56%)	208.00 (50.36%)	137.00 (60.35%)	
	≥1/week	2092.00 (58.05%)	1001.00 (62.48%)	796.00 (58.44%)	205.00 (49.64%)	90.00 (39.65%)	
Physical_activity, *n* (*p*%)							< 0.001
	High	2926.00 (81.19%)	1361.00 (84.96%)	1126.00 (82.67%)	304.00 (73.61%)	135.00 (59.47%)	
	Low	678.00 (18.81%)	241.00 (15.04%)	236.00 (17.33%)	109.00 (26.39%)	92.00 (40.53%)	
Diabetes, *n* (*p*%)							< 0.001
	No	3155.00 (87.54%)	1433.00 (89.45%)	1195.00 (87.74%)	344.00 (83.29%)	183.00 (80.62%)	
	Yes	449.00 (12.46%)	169.00 (10.55%)	167.00 (12.26%)	69.00 (16.71%)	44.00 (19.38%)	
Hyperlipidemia, *n* (*p*%)							< 0.001
	No	2157.00 (59.85%)	1035.00 (64.61%)	784.00 (57.56%)	235.00 (56.90%)	103.00 (45.37%)	
	Yes	1447.00 (40.15%)	567.00 (35.39%)	578.00 (42.44%)	178.00 (43.10%)	124.00 (54.63%)	
Hypertension, *n* (*p*%)							0.006
	No	1859.00 (51.58%)	876.00 (54.68%)	681.00 (50.00%)	193.00 (46.73%)	109.00 (48.02%)	
	Yes	1745.00 (48.42%)	726.00 (45.32%)	681.00 (50.00%)	220.00 (53.27%)	118.00 (51.98%)	
Cancer, *n* (*p*%)							< 0.001
	No	3155.00 (87.54%)	1435.00 (89.58%)	1185.00 (87.00%)	341.00 (82.57%)	194.00 (85.46%)	
	Yes	449.00 (12.46%)	167.00 (10.42%)	177.00 (13.00%)	72.00 (17.43%)	33.00 (14.54%)	
Stroke, *n* (*p*%)							0.052
	No	3446.00 (95.62%)	1543.00 (96.32%)	1303.00 (95.67%)	389.00 (94.19%)	211.00 (92.95%)	
	Yes	158.00 (4.38%)	59.00 (3.68%)	59.00 (4.33%)	24.00 (5.81%)	16.00 (7.05%)	
Chronic lung disease, *n* (*p*%)							< 0.001
	No	3412.00 (94.67%)	1547.00 (96.57%)	1289.00 (94.64%)	372.00 (90.07%)	204.00 (89.87%)	
	Yes	192.00 (5.33%)	55.00 (3.43%)	73.00 (5.36%)	41.00 (9.93%)	23.00 (10.13%)	
OA, *n* (*p*%)							< 0.001
	No	3234.00 (89.73%)	1495.00 (93.32%)	1212.00 (88.99%)	347.00 (84.02%)	180.00 (79.30%)	
	Yes	370.00 (10.27%)	107.00 (6.68%)	150.00 (11.01%)	66.00 (15.98%)	47.00 (20.70%)	

### 3.2 Multi‐Trajectories of Sleep Disturbance and Depressive Symptoms

**FIGURE 3:**
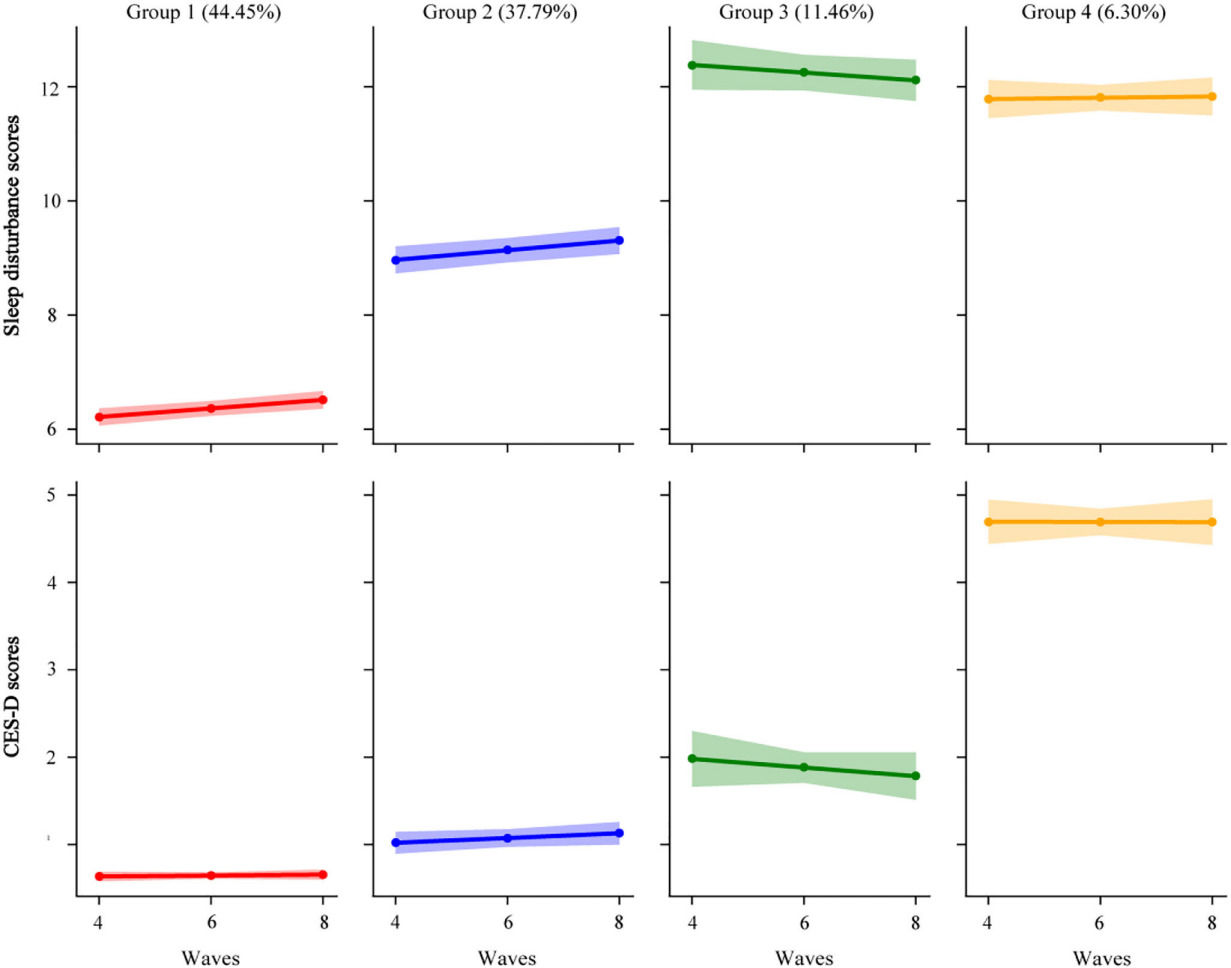
Multi‐trajectories of sleep disturbance and depressive symptoms from ELSA.

GBMTM identified four multi‐trajectory groups (Figure 3):

*Group 1*: Persistently good sleep/no depressive symptoms (44.45%)

*Group 2*: Persistently moderate sleep disturbance/low depressive symptoms (37.79%)

*Group 3*: Persistently poor sleep/moderate depressive symptoms (11.46%)

*Group 4*: Persistently poor sleep/persistently high depressive symptoms (6.30%)

Distinct and stable differences in sleep disturbance and depressive symptoms were observed across Waves 4, 6, and 8 (Figure 3). Detailed modeling information is presented in Tables S2–S5.

### 3.3 Multi‐Trajectories and Incident OA

In logistic regression analyses, joint trajectory membership was significantly associated with incident OA (Table 2). In the fully adjusted model (Model 3), compared with the persistently good sleep/no depressive symptoms group, the odds of incident OA were higher in the persistently moderate sleep disturbance/low depressive symptoms group (OR = 1.56, 95% CI 1.20–2.03, *p* = 0.001), the persistently poor sleep/moderate depressive symptoms group (OR = 2.14, 95% CI 1.52–3.02, *p < *0.001), and highest in the persistently poor sleep/persistently high depressive symptoms group (OR = 2.85, 95% CI 1.90–4.28, *p < *0.001). Overall, a clear gradient was observed, with progressively worse long‐term sleep and depressive symptom patterns associated with higher odds of incident OA.

**TABLE 2:** Correlation between multi‐trajectories of sleep disturbance and depressive symptoms and the risk of OA.

Variables Group	Model 1		Model 2		Model 3	
	OR (95%CI)	*p*	OR (95%CI)	*p*	OR (95%CI)	*p*
1	1.00 (Reference)		1.00 (Reference)		1.00 (Reference)	
2	1.73 (1.33–2.24)	< 0.001	1.63 (1.25–2.12)	< 0.001	1.56 (1.20–2.03)	0.001
3	2.66 (1.91–3.69)	< 0.001	2.36 (1.69–3.29)	< 0.001	2.14 (1.52–3.02)	< 0.001
4	3.65 (2.50–5.31)	< 0.001	3.21 (2.17–4.75)	< 0.001	2.85 (1.90–4.28)	< 0.001

*Note*: Model1: crude. Model2: Adjust: age, gender, race, marital, education, income. Model3: Adjust: age, gender, race, marital, education, income, drink, smoke, physical activity, BMI, hyperlipidemia, hypertension, diabetes, cancer, chronic lung disease, stroke.

Abbreviations: CI, confidence interval; OR, odds ratio.

### 3.4 Subgroup Analyses

**FIGURE 4:**
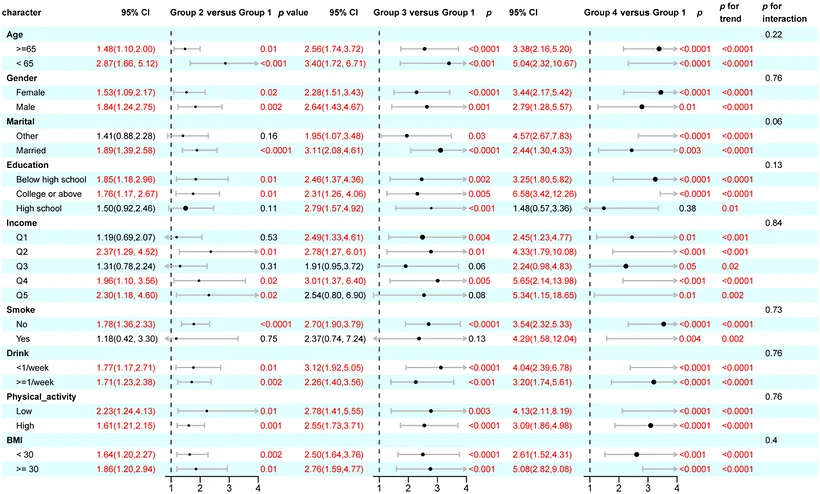
The association between multi‐trajectories of sleep disturbance and depressive symptoms and OA in different subgroups.

Subgroup analyses yielded broadly consistent estimates across strata (Figure 4). For Group 4 versus Group 1, the OR was 5.04 (95% CI 2.32–10.67) among participants aged < 65 years and 3.38 (95% CI 2.16–5.20) among those aged ≥ 65 years. However, the age interaction was not statistically significant (*p* for interaction = 0.22). Among participants aged < 65 years, incident OA occurred in 19/446 (4.26%) in Group 1 and 13/71 (18.31%) in Group 4. Corresponding values at age ≥ 65 years were 88/1,156 (7.61%) and 34/156 (21.79%). The absolute risk differences for Group 4 versus Group 1 were therefore similar: 14.05 and 14.18 percentage points, respectively. The numerically larger relative OR in younger participants thus coexisted with nearly identical absolute risk differences. Other subgroup estimates are shown in Figure 4, and no statistically significant interactions were detected (all *p* for interaction > 0.05).

## 4 Discussion

Using data from a large, nationally representative aging cohort, this study examined joint longitudinal trajectories of sleep disturbance and depressive symptoms and their association with incident OA. The main finding was that persistent co‐occurrence of poor sleep and elevated depressive symptoms was associated with subsequent OA. Compared with participants who maintained persistently good sleep and minimal depressive symptoms, those following a “persistently poor sleep/persistently high depressive symptoms” trajectory had markedly higher odds of incident OA. These findings underscore the relevance of sleep and mental health for joint health and identify candidate markers for risk stratification and future causal evaluation.

The age‐stratified pattern warrants cautious interpretation. Although the Group 4 OR was numerically larger below age 65 years, the absolute risk difference was almost identical across age strata and the interaction was not statistically significant. The younger Group 4 estimate was also less precise because this stratum contained only 71 participants and 13 events. A lower OA incidence in the younger reference group can yield a larger relative OR even when the absolute contrast is similar. We therefore interpret this subgroup finding as exploratory rather than evidence that younger adults are intrinsically more susceptible.

Prior evidence has linked either sleep problems or depression to OA outcomes, but most studies have adopted a single‐exposure perspective. Prospective cohorts and meta‐analyses have reported associations of insomnia, short sleep, and poor sleep quality with incident OA and greater pain burden, with Mendelian randomization studies further supporting potential causal roles of insomnia and short sleep in OA risk (Chen et al. 2025; Long et al. 2025; Ni et al. 2022). In parallel, clinical and population‐based studies consistently show that depression is related to worse OA pain, functional limitation, and overall disease burden (Wang and Ni 2022; Zhang et al. 2024). However, sleep and depression are rarely static or independent in later life; many studies have not captured how they co‐evolve over time, nor how their combined trajectories shape longer‐term OA risk (Song et al. 2020; Wang et al. 2025; Xia et al. 2025). By identifying heterogeneous joint patterns of sleep disturbance and depressive symptoms and linking these patterns prospectively to incident OA, our results extend prior work beyond single‐time‐point or single‐exposure approaches. This trajectory‐based framework aligns with the growing view that OA is influenced by multisystem, modifiable factors rather than being solely a “joint‐centered” disorder (Hunter et al. 2020).

The observed gradient across joint trajectory groups is biologically plausible and may reflect shared and mutually reinforcing pathways. First, both sleep disturbance and depressive symptoms are associated with chronic low‐grade inflammation and upregulated pro‐inflammatory cytokines (Engert and Besedovsky 2025; Veler 2023; Yin et al. 2024), which have been implicated in synovitis, cartilage matrix imbalance, and pain amplification in OA (De Roover et al. 2023; Sanchez‐Lopez et al. 2022; Wijesinghe et al. 2024). Persistent exposure to both conditions may therefore increase systemic inflammatory load. Second, sleep problems and depression can disrupt hypothalamic–pituitary–adrenal (HPA) axis function and cortisol rhythms (de Leeuw et al. 2023; Dressle et al. 2022); sustained stress‐related HPA dysregulation may promote inflammation, metabolic disturbances, and pain sensitization (Lee et al. 2024; Mbiydzenyuy and Qulu 2024; Rösch et al. 2025), potentially accelerating OA pathophysiology. Third, impaired sleep and depressive symptoms may contribute to central sensitization and altered pain processing (Bordvik et al. 2024; Chang et al. 2022; Dahmani et al. 2023; Kourbanova et al. 2022; Li et al. 2025; Saxer et al. 2024), while fatigue, reduced activity, and avoidance behaviors can increase mechanical load and weight gain (van Dongen et al. 2025; Vitiello et al. 2021), reinforcing a maladaptive bio‐behavioral cycle.

These findings have clinical and public health implications. Incorporating sleep and mental health into OA risk assessment may improve early identification of individuals at elevated risk, particularly among older adults with early joint symptoms who have not yet received an OA diagnosis. Moreover, in the absence of widely available disease‐modifying OA drugs (DMOADs) and with current care focused largely on symptom management, prevention research on potentially modifiable upstream factors remains important (Brandt et al. 2025; Jenei‐Lanzl et al. 2025). Randomized trials and recent reviews indicate that cognitive behavioral therapy for insomnia (CBT‐I) improves sleep and can also reduce depressive symptoms, with durable effects (Furukawa et al. 2024; Tamm et al. 2025; Vitiello et al. 2021). Integrating evidence‐based sleep interventions such as CBT‐I into routine ageing health programs, especially for individuals with persistent sleep–depression comorbidity trajectories, may offer dual benefits for sleep and emotional health. Whether these interventions reduce future OA risk requires direct evaluation.

This study has several strengths, including a large, high‐quality, nationally representative cohort, a longitudinal design supporting temporal ordering, and the use of GBMTM to capture long‐term heterogeneity beyond single measurements. Limitations should also be considered. First, sleep disturbance, depressive symptoms, and OA were primarily self‐reported, which may introduce recall bias and misclassification; non‐differential error could attenuate associations. Second, despite adjustment for multiple confounders, residual confounding and reverse causation cannot be fully excluded, as subclinical joint pathology or chronic pain may influence sleep and mood prior to OA diagnosis. Third, trajectory modeling is a latent‐class approach; selection of group number and shape depends on modeling assumptions and researcher judgement, and comparability of trajectories across studies may be limited (Hetherington et al. 2020). Fourth, although ELSA is broadly representative of adults aged ≥50 years in England, associations may differ across populations with different ethnic composition, cultural contexts, and healthcare systems, warranting replication in other cohorts. Finally, attrition and survivor bias are common in aging studies; participants with higher symptom burden may be more likely to drop out or die, potentially underestimating true associations (Heid et al. 2021; Hernandez et al. 2024). Future research incorporating objective sleep measures, clinical/imaging‐based OA ascertainment, and stronger causal inference approaches across diverse populations will help to validate and extend these findings.

## 5 Conclusion

In summary, persistent co‐occurring sleep disturbance and depressive symptoms were associated with higher odds of incident OA in middle‐aged and older adults. The graded associations support considering sleep and mental health in OA risk assessment. Because the study was observational, the findings do not establish that modifying these factors will prevent OA. Future intervention and causal‐inference studies should test whether these trajectories represent modifiable determinants or risk markers.

## Author Contributions

**Shufang Deng**: conceptualization, formal analysis, visualization, funding acquisition, writing – original draft. **Zeping Chen**: data curation, methodology, writing – original draft. **Wei Zhao**: methodology, writing – original draft. **Zhaoheng Chen**: visualization, writing – original draft. **Guimin Zhang**: validation, writing – original draft. **Guimin Zhang**: methodology, writing – original draft. **Peijuan Li**: visualization, writing – original draft. **Ningyuan Lai**: conceptualization, writing – original draft. **Rui Xie**: formal analysis, supervision, writing – review and editing.

## Funding

This work was supported by a grant from the Sichuan Provincial Administration of Traditional Chinese Medicine (2024MS406), and Chengdu University of Traditional Chinese Medicine 2024 Annual Educational and Teaching Reform Project (JGJD202451).

## Ethics Statement

The English Longitudinal Study of Ageing received ethical approval from the London Multicenter Research Ethics Committee (MREC/01/2/91). All participants signed the informed consent document. The authors assert that all procedures contributing to this work comply with the ethical standards of the relevant national and institutional committees on human experimentation and with the Helsinki Declaration of 1975, as revised in 2008.

## Conflicts of Interest

The authors declare no conflicts of interest.

## Supporting information




**Supplementary Material**: brb371788‐sup‐0001‐SuppMat.docx

## Data Availability

The dataset used in this study is publicly accessible through the ELSA.
