# Supplementary material for: Multi‐Trajectories of Sleep Disturbance and Depressive Symptoms and Incident Osteoarthritis: Evidence From the English Longitudinal Study of Ageing
Source: Brain Behav. 2026 Sep 23;16(9):e71788. doi: 10.1002/brb3.71788 (PMC13601843; doi:10.1002/brb3.71788)
Supplement: Supplementary file 1 [file BRB3-16-e71788-s001.docx]

Supplementary Material

**Multi-trajectories of sleep disturbance and depressive symptoms and incident osteoarthritis: evidence from the English Longitudinal Study of Ageing**

**Supplemental Tables S1.** The numbers (percentages) of participants with missing covariates.

**Supplemental Tables S2.** Evaluation Indicators of the fitting effect of multiple models.

**Supplemental Tables S3.** Multiple model trajectory group evaluation indicators.

**Supplemental Tables S4.** Trajectory parameter estimation for the model.

**Supplemental Tables S5**. The best trajectory model applicability.

**Supplemental Tables S1.** The numbers (percentages) of participants with missing covariates.

| **Covariate** | **Number** | **Percentage (%)** |
| --- | --- | --- |
| BMI | 269 | 7.5 |
| Education | 255 | 7.1 |
| Income | 4 | 0.1 |
| Smoke | 29 | 0.5 |
| Alcohol consumption | 297 | 8.2 |

**Supplemental Tables S2.** Evaluation Indicators of the fitting effect of multiple models.

| **Model ID** | ***Log(LL)*** | ***BIC*** | ***AIC*** | ***E_k_*** |
| --- | --- | --- | --- | --- |
| M1[11][11] | -44885.874 | -44930.918 | -44896.874 | 0.868 |
| M2[22][22] | -44882.582 | -44944.006 | -44897.582 | 0.869 |
| M3[33][33] | -44882.582 | -44960.385 | -44901.582 | 0.869 |
| M4[111][111] | -43844.845 | -43910.363 | -43860.845 | 0.845 |
| M5[222][222] | -43831.999 | -43922.086 | -43853.999 | 0.845 |
| M6[333][333] | -43831.999 | -43946.656 | -43859.999 | 0.845 |
| M7[1111][1111] | -43597.088 | -43683.081 | -43618.088 | 0.770 |
| M8[2222][2222] | -43583.970 | -43702.722 | -43612.970 | 0.772 |
| M9[3333][3333] | -43583.970 | -43735.482 | -43620.970 | 0.772 |

**Supplemental Tables S3.** Multiple model trajectory group evaluation indicators.

| **Model ID** | **Class** | ***OCC*** | ***AvepP*** | ***P*** | ***π*** |
| --- | --- | --- | --- | --- | --- |
| M1[11][11] | Class1 | 11.067 | 97.277 | 76.637 | 76.350 |
| M1[11][11] | Class2 | 38.677 | 92.296 | 23.363 | 23.650 |
| M2[22][22] | Class1 | 10.962 | 97.272 | 76.804 | 76.489 |
| M2[22][22] | Class2 | 39.132 | 92.324 | 23.196 | 23.511 |
| M3[33][33] | Class1 | 10.962 | 97.273 | 76.804 | 76.489 |
| M3[33][33] | Class2 | 39.129 | 92.324 | 23.196 | 23.511 |
| M4[111][111] | Class1 | 11.172 | 94.819 | 62.486 | 62.094 |
| M4[111][111] | Class2 | 17.525 | 88.321 | 29.967 | 30.144 |
| M4[111][111] | Class3 | 186.082 | 93.997 | 7.547 | 7.762 |
| M5[222][222] | Class1 | 11.064 | 94.743 | 62.431 | 61.960 |
| M5[222][222] | Class2 | 17.807 | 88.476 | 29.828 | 30.126 |
| M5[222][222] | Class3 | 180.195 | 93.934 | 7.741 | 7.914 |
| M6[333][333] | Class1 | 11.065 | 94.743 | 62.431 | 61.960 |
| M6[333][333] | Class2 | 17.808 | 88.476 | 29.828 | 30.126 |
| M6[333][333] | Class3 | 180.173 | 93.933 | 7.741 | 7.914 |
| M7[1111][1111] | Class1 | 10.176 | 88.911 | 44.451 | 44.070 |
| M7[1111][1111] | Class2 | 7.786 | 82.362 | 37.791 | 37.489 |
| M7[1111][1111] | Class3 | 40.573 | 84.831 | 11.459 | 12.114 |
| M7[1111][1111] | Class4 | 189.546 | 92.755 | 6.299 | 6.327 |
| M8[2222][2222] | Class1 | 10.093 | 88.969 | 44.922 | 44.417 |
| M8[2222][2222] | Class2 | 7.959 | 82.622 | 37.569 | 37.396 |
| M8[2222][2222] | Class3 | 40.865 | 84.514 | 11.182 | 11.781 |
| M8[2222][2222] | Class4 | 197.918 | 93.125 | 6.326 | 6.405 |
| M9[3333][3333] | Class1 | 10.093 | 88.970 | 44.922 | 44.417 |
| M9[3333][3333] | Class2 | 7.959 | 82.621 | 37.569 | 37.396 |
| M9[3333][3333] | Class3 | 40.866 | 84.515 | 11.182 | 11.782 |
| M9[3333][3333] | Class4 | 197.915 | 93.125 | 6.326 | 6.405 |

**Supplemental Tables S4.** Trajectory parameter estimation for the model.

| **Model ID** | **Traj_Y** | ***Parameter*** | ***Class*** | ***β*** | ***SE*** | ***t*** | ***P*** |
| --- | --- | --- | --- | --- | --- | --- | --- |
| M1[11][11] | sleep disturbance score | Intercept | Class1 | 7.173 | 0.078 | 92.188 | <0.001 |
| M1[11][11] | sleep disturbance score | Linear | Class1 | 0.152 | 0.033 | 4.657 | <0.001 |
| M1[11][11] | sleep disturbance score | Intercept | Class2 | 11.670 | 0.139 | 83.841 | <0.001 |
| M1[11][11] | sleep disturbance score | Linear | Class2 | -0.009 | 0.062 | -0.144 | 0.885 |
| M1[11][11] | depressive symptoms score | Intercept | Class1 | 0.463 | 0.041 | 11.298 | <0.001 |
| M1[11][11] | depressive symptoms score | Linear | Class1 | 0.033 | 0.019 | 1.757 | 0.079 |
| M1[11][11] | depressive symptoms score | Intercept | Class2 | 2.691 | 0.094 | 28.656 | <0.001 |
| M1[11][11] | depressive symptoms score | Linear | Class2 | -0.023 | 0.036 | -0.640 | 0.522 |
| M2[22][22] | sleep disturbance score | Intercept | Class1 | 7.457 | 0.203 | 36.696 | <0.001 |
| M2[22][22] | sleep disturbance score | Linear | Class1 | -0.181 | 0.227 | -0.798 | 0.425 |
| M2[22][22] | sleep disturbance score | Quadratic | Class1 | 0.083 | 0.056 | 1.481 | 0.139 |
| M2[22][22] | sleep disturbance score | Intercept | Class2 | 11.448 | 0.375 | 30.516 | <0.001 |
| M2[22][22] | sleep disturbance score | Linear | Class2 | 0.264 | 0.425 | 0.620 | 0.535 |
| M2[22][22] | sleep disturbance score | Quadratic | Class2 | -0.068 | 0.105 | -0.647 | 0.518 |
| M2[22][22] | depressive symptoms score | Intercept | Class1 | 0.683 | 0.116 | 5.911 | <0.001 |
| M2[22][22] | depressive symptoms score | Linear | Class1 | -0.230 | 0.131 | -1.760 | 0.078 |
| M2[22][22] | depressive symptoms score | Quadratic | Class1 | 0.066 | 0.032 | 2.033 | 0.042 |
| M2[22][22] | depressive symptoms score | Intercept | Class2 | 2.597 | 0.221 | 11.728 | <0.001 |
| M2[22][22] | depressive symptoms score | Linear | Class2 | 0.100 | 0.251 | 0.399 | 0.690 |
| M2[22][22] | depressive symptoms score | Quadratic | Class2 | -0.031 | 0.062 | -0.495 | 0.621 |
| M3[33][33] | sleep disturbance score | Intercept | Class1 | 7.201 | 2147.256 | 0.003 | 0.997 |
| M3[33][33] | sleep disturbance score | Linear | Class1 | 0.289 | 3936.636 | 0.000 | 1.000 |
| M3[33][33] | sleep disturbance score | Quadratic | Class1 | -0.173 | 2147.256 | -0.000 | 1.000 |
| M3[33][33] | sleep disturbance score | Cubic | Class1 | 0.043 | 357.876 | 0.000 | 1.000 |
| M3[33][33] | sleep disturbance score | Intercept | Class2 | 11.363 | 4042.885 | 0.003 | 0.998 |
| M3[33][33] | sleep disturbance score | Linear | Class2 | 0.419 | 7411.956 | 0.000 | 1.000 |
| M3[33][33] | sleep disturbance score | Quadratic | Class2 | -0.153 | 4042.885 | -0.000 | 1.000 |
| M3[33][33] | sleep disturbance score | Cubic | Class2 | 0.014 | 673.814 | 0.000 | 1.000 |
| M3[33][33] | depressive symptoms score | Intercept | Class1 | 0.456 | 299.163 | 0.002 | 0.999 |
| M3[33][33] | depressive symptoms score | Linear | Class1 | 0.186 | 548.465 | 0.000 | 1.000 |
| M3[33][33] | depressive symptoms score | Quadratic | Class1 | -0.161 | 299.163 | -0.001 | 1.000 |
| M3[33][33] | depressive symptoms score | Cubic | Class1 | 0.038 | 49.860 | 0.001 | 0.999 |
| M3[33][33] | depressive symptoms score | Intercept | Class2 | 2.546 | 615.797 | 0.004 | 0.997 |
| M3[33][33] | depressive symptoms score | Linear | Class2 | 0.193 | 1128.962 | 0.000 | 1.000 |
| M3[33][33] | depressive symptoms score | Quadratic | Class2 | -0.081 | 615.797 | -0.000 | 1.000 |
| M3[33][33] | depressive symptoms score | Cubic | Class2 | 0.008 | 102.633 | 0.000 | 1.000 |
| M4[111][111] | sleep disturbance score | Intercept | Class1 | 6.604 | 0.079 | 83.771 | <0.001 |
| M4[111][111] | sleep disturbance score | Linear | Class1 | 0.171 | 0.034 | 4.978 | <0.001 |
| M4[111][111] | sleep disturbance score | Intercept | Class2 | 10.601 | 0.125 | 84.752 | <0.001 |
| M4[111][111] | sleep disturbance score | Linear | Class2 | 0.039 | 0.053 | 0.747 | 0.455 |
| M4[111][111] | sleep disturbance score | Intercept | Class3 | 12.116 | 0.222 | 54.484 | <0.001 |
| M4[111][111] | sleep disturbance score | Linear | Class3 | -0.056 | 0.101 | -0.553 | 0.580 |
| M4[111][111] | depressive symptoms score | Intercept | Class1 | 0.367 | 0.042 | 8.768 | <0.001 |
| M4[111][111] | depressive symptoms score | Linear | Class1 | 0.033 | 0.019 | 1.702 | 0.089 |
| M4[111][111] | depressive symptoms score | Intercept | Class2 | 1.342 | 0.074 | 18.047 | <0.001 |
| M4[111][111] | depressive symptoms score | Linear | Class2 | 0.024 | 0.033 | 0.721 | 0.471 |
| M4[111][111] | depressive symptoms score | Intercept | Class3 | 4.605 | 0.166 | 27.739 | <0.001 |
| M4[111][111] | depressive symptoms score | Linear | Class3 | -0.099 | 0.074 | -1.340 | 0.180 |
| M5[222][222] | sleep disturbance score | Intercept | Class1 | 6.850 | 0.213 | 32.234 | <0.001 |
| M5[222][222] | sleep disturbance score | Linear | Class1 | -0.131 | 0.239 | -0.547 | 0.585 |
| M5[222][222] | sleep disturbance score | Quadratic | Class1 | 0.076 | 0.059 | 1.280 | 0.201 |
| M5[222][222] | sleep disturbance score | Intercept | Class2 | 10.845 | 0.322 | 33.687 | <0.001 |
| M5[222][222] | sleep disturbance score | Linear | Class2 | -0.262 | 0.360 | -0.727 | 0.467 |
| M5[222][222] | sleep disturbance score | Quadratic | Class2 | 0.075 | 0.089 | 0.838 | 0.402 |
| M5[222][222] | sleep disturbance score | Intercept | Class3 | 11.171 | 0.601 | 18.584 | <0.001 |
| M5[222][222] | sleep disturbance score | Linear | Class3 | 1.050 | 0.681 | 1.541 | 0.123 |
| M5[222][222] | sleep disturbance score | Quadratic | Class3 | -0.274 | 0.168 | -1.626 | 0.104 |
| M5[222][222] | depressive symptoms score | Intercept | Class1 | 0.488 | 0.118 | 4.151 | <0.001 |
| M5[222][222] | depressive symptoms score | Linear | Class1 | -0.115 | 0.133 | -0.865 | 0.387 |
| M5[222][222] | depressive symptoms score | Quadratic | Class1 | 0.037 | 0.033 | 1.128 | 0.259 |
| M5[222][222] | depressive symptoms score | Intercept | Class2 | 1.893 | 0.189 | 10.036 | <0.001 |
| M5[222][222] | depressive symptoms score | Linear | Class2 | -0.649 | 0.213 | -3.054 | 0.002 |
| M5[222][222] | depressive symptoms score | Quadratic | Class2 | 0.168 | 0.052 | 3.211 | 0.001 |
| M5[222][222] | depressive symptoms score | Intercept | Class3 | 3.287 | 0.388 | 8.460 | <0.001 |
| M5[222][222] | depressive symptoms score | Linear | Class3 | 1.448 | 0.428 | 3.382 | 0.001 |
| M5[222][222] | depressive symptoms score | Quadratic | Class3 | -0.387 | 0.106 | -3.659 | <0.001 |
| M6[333][333] | sleep disturbance score | Intercept | Class1 | 6.542 | 749.618 | 0.009 | 0.993 |
| M6[333][333] | sleep disturbance score | Linear | Class1 | 0.435 | 1374.313 | 0.000 | 1.000 |
| M6[333][333] | sleep disturbance score | Quadratic | Class1 | -0.233 | 749.617 | -0.000 | 1.000 |
| M6[333][333] | sleep disturbance score | Cubic | Class1 | 0.051 | 124.936 | 0.000 | 1.000 |
| M6[333][333] | sleep disturbance score | Intercept | Class2 | 10.300 | 460.840 | 0.022 | 0.982 |
| M6[333][333] | sleep disturbance score | Linear | Class2 | 0.736 | 844.873 | 0.001 | 0.999 |
| M6[333][333] | sleep disturbance score | Quadratic | Class2 | -0.470 | 460.841 | -0.001 | 0.999 |
| M6[333][333] | sleep disturbance score | Cubic | Class2 | 0.091 | 76.807 | 0.001 | 0.999 |
| M6[333][333] | sleep disturbance score | Intercept | Class3 | 11.626 | 605.799 | 0.019 | 0.985 |
| M6[333][333] | sleep disturbance score | Linear | Class3 | 0.215 | 1110.632 | 0.000 | 1.000 |
| M6[333][333] | sleep disturbance score | Quadratic | Class3 | 0.182 | 605.800 | 0.000 | 1.000 |
| M6[333][333] | sleep disturbance score | Cubic | Class3 | -0.076 | 100.967 | -0.001 | 0.999 |
| M6[333][333] | depressive symptoms score | Intercept | Class1 | 0.242 | 31.246 | 0.008 | 0.994 |
| M6[333][333] | depressive symptoms score | Linear | Class1 | 0.337 | 57.281 | 0.006 | 0.995 |
| M6[333][333] | depressive symptoms score | Quadratic | Class1 | -0.209 | 31.250 | -0.007 | 0.995 |
| M6[333][333] | depressive symptoms score | Cubic | Class1 | 0.041 | 5.207 | 0.008 | 0.994 |
| M6[333][333] | depressive symptoms score | Intercept | Class2 | 1.480 | 238.208 | 0.006 | 0.995 |
| M6[333][333] | depressive symptoms score | Linear | Class2 | 0.107 | 436.715 | 0.000 | 1.000 |
| M6[333][333] | depressive symptoms score | Quadratic | Class2 | -0.244 | 238.208 | -0.001 | 0.999 |
| M6[333][333] | depressive symptoms score | Cubic | Class2 | 0.069 | 39.701 | 0.002 | 0.999 |
| M6[333][333] | depressive symptoms score | Intercept | Class3 | 3.733 | 1368.549 | 0.003 | 0.998 |
| M6[333][333] | depressive symptoms score | Linear | Class3 | 0.630 | 2509.007 | 0.000 | 1.000 |
| M6[333][333] | depressive symptoms score | Quadratic | Class3 | 0.060 | 1368.550 | 0.000 | 1.000 |
| M6[333][333] | depressive symptoms score | Cubic | Class3 | -0.074 | 228.092 | -0.000 | 1.000 |
| M7[1111][1111] | sleep disturbance score | Intercept | Class1 | 6.066 | 0.101 | 59.902 | <0.001 |
| M7[1111][1111] | sleep disturbance score | Linear | Class1 | 0.150 | 0.040 | 3.730 | <0.001 |
| M7[1111][1111] | sleep disturbance score | Intercept | Class2 | 8.798 | 0.148 | 59.427 | <0.001 |
| M7[1111][1111] | sleep disturbance score | Linear | Class2 | 0.169 | 0.050 | 3.363 | 0.001 |
| M7[1111][1111] | sleep disturbance score | Intercept | Class3 | 12.549 | 0.260 | 48.321 | <0.001 |
| M7[1111][1111] | sleep disturbance score | Linear | Class3 | -0.139 | 0.103 | -1.356 | 0.175 |
| M7[1111][1111] | sleep disturbance score | Intercept | Class4 | 11.773 | 0.236 | 49.818 | <0.001 |
| M7[1111][1111] | sleep disturbance score | Linear | Class4 | 0.023 | 0.107 | 0.215 | 0.830 |
| M7[1111][1111] | depressive symptoms score | Intercept | Class1 | 0.319 | 0.051 | 6.307 | <0.001 |
| M7[1111][1111] | depressive symptoms score | Linear | Class1 | 0.016 | 0.023 | 0.691 | 0.489 |
| M7[1111][1111] | depressive symptoms score | Intercept | Class2 | 0.805 | 0.075 | 10.779 | <0.001 |
| M7[1111][1111] | depressive symptoms score | Linear | Class2 | 0.068 | 0.031 | 2.154 | 0.031 |
| M7[1111][1111] | depressive symptoms score | Intercept | Class3 | 2.063 | 0.213 | 9.665 | <0.001 |
| M7[1111][1111] | depressive symptoms score | Linear | Class3 | -0.105 | 0.094 | -1.110 | 0.267 |
| M7[1111][1111] | depressive symptoms score | Intercept | Class4 | 4.697 | 0.224 | 20.969 | <0.001 |
| M7[1111][1111] | depressive symptoms score | Linear | Class4 | -0.002 | 0.106 | -0.021 | 0.983 |
| M8[2222][2222] | sleep disturbance score | Intercept | Class1 | 6.309 | 0.246 | 25.671 | <0.001 |
| M8[2222][2222] | sleep disturbance score | Linear | Class1 | -0.132 | 0.273 | -0.486 | 0.627 |
| M8[2222][2222] | sleep disturbance score | Quadratic | Class1 | 0.071 | 0.067 | 1.057 | 0.290 |
| M8[2222][2222] | sleep disturbance score | Intercept | Class2 | 9.278 | 0.316 | 29.321 | <0.001 |
| M8[2222][2222] | sleep disturbance score | Linear | Class2 | -0.360 | 0.325 | -1.107 | 0.268 |
| M8[2222][2222] | sleep disturbance score | Quadratic | Class2 | 0.129 | 0.080 | 1.614 | 0.106 |
| M8[2222][2222] | sleep disturbance score | Intercept | Class3 | 12.111 | 0.554 | 21.861 | <0.001 |
| M8[2222][2222] | sleep disturbance score | Linear | Class3 | 0.395 | 0.624 | 0.633 | 0.527 |
| M8[2222][2222] | sleep disturbance score | Quadratic | Class3 | -0.125 | 0.153 | -0.818 | 0.414 |
| M8[2222][2222] | sleep disturbance score | Intercept | Class4 | 10.884 | 0.635 | 17.131 | <0.001 |
| M8[2222][2222] | sleep disturbance score | Linear | Class4 | 1.092 | 0.718 | 1.521 | 0.128 |
| M8[2222][2222] | sleep disturbance score | Quadratic | Class4 | -0.272 | 0.177 | -1.535 | 0.125 |
| M8[2222][2222] | depressive symptoms score | Intercept | Class1 | 0.371 | 0.141 | 2.627 | 0.009 |
| M8[2222][2222] | depressive symptoms score | Linear | Class1 | -0.047 | 0.160 | -0.292 | 0.770 |
| M8[2222][2222] | depressive symptoms score | Quadratic | Class1 | 0.016 | 0.040 | 0.405 | 0.686 |
| M8[2222][2222] | depressive symptoms score | Intercept | Class2 | 1.325 | 0.187 | 7.092 | <0.001 |
| M8[2222][2222] | depressive symptoms score | Linear | Class2 | -0.531 | 0.197 | -2.692 | 0.007 |
| M8[2222][2222] | depressive symptoms score | Quadratic | Class2 | 0.147 | 0.048 | 3.057 | 0.002 |
| M8[2222][2222] | depressive symptoms score | Intercept | Class3 | 2.102 | 0.404 | 5.202 | <0.001 |
| M8[2222][2222] | depressive symptoms score | Linear | Class3 | -0.231 | 0.433 | -0.533 | 0.594 |
| M8[2222][2222] | depressive symptoms score | Quadratic | Class3 | 0.045 | 0.104 | 0.427 | 0.669 |
| M8[2222][2222] | depressive symptoms score | Intercept | Class4 | 3.517 | 0.454 | 7.743 | <0.001 |
| M8[2222][2222] | depressive symptoms score | Linear | Class4 | 1.469 | 0.502 | 2.925 | 0.003 |
| M8[2222][2222] | depressive symptoms score | Quadratic | Class4 | -0.382 | 0.125 | -3.056 | 0.002 |
| M9[3333][3333] | sleep disturbance score | Intercept | Class1 | 5.991 | 2297.423 | 0.003 | 0.998 |
| M9[3333][3333] | sleep disturbance score | Linear | Class1 | 0.450 | 4211.944 | 0.000 | 1.000 |
| M9[3333][3333] | sleep disturbance score | Quadratic | Class1 | -0.247 | 2297.424 | -0.000 | 1.000 |
| M9[3333][3333] | sleep disturbance score | Cubic | Class1 | 0.053 | 382.904 | 0.000 | 1.000 |
| M9[3333][3333] | sleep disturbance score | Intercept | Class2 | 8.760 | 803.445 | 0.011 | 0.991 |
| M9[3333][3333] | sleep disturbance score | Linear | Class2 | 0.588 | 1472.983 | 0.000 | 1.000 |
| M9[3333][3333] | sleep disturbance score | Quadratic | Class2 | -0.388 | 803.444 | -0.000 | 1.000 |
| M9[3333][3333] | sleep disturbance score | Cubic | Class2 | 0.086 | 133.908 | 0.001 | 0.999 |
| M9[3333][3333] | sleep disturbance score | Intercept | Class3 | 11.812 | 4152.730 | 0.003 | 0.998 |
| M9[3333][3333] | sleep disturbance score | Linear | Class3 | 0.944 | 7613.338 | 0.000 | 1.000 |
| M9[3333][3333] | sleep disturbance score | Quadratic | Class3 | -0.424 | 4152.730 | -0.000 | 1.000 |
| M9[3333][3333] | sleep disturbance score | Cubic | Class3 | 0.050 | 692.122 | 0.000 | 1.000 |
| M9[3333][3333] | sleep disturbance score | Intercept | Class4 | 11.517 | 448.131 | 0.026 | 0.979 |
| M9[3333][3333] | sleep disturbance score | Linear | Class4 | -0.068 | 821.573 | -0.000 | 1.000 |
| M9[3333][3333] | sleep disturbance score | Quadratic | Class4 | 0.361 | 448.131 | 0.001 | 0.999 |
| M9[3333][3333] | sleep disturbance score | Cubic | Class4 | -0.105 | 74.688 | -0.001 | 0.999 |
| M9[3333][3333] | depressive symptoms score | Intercept | Class1 | 0.113 | 92.068 | 0.001 | 0.999 |
| M9[3333][3333] | depressive symptoms score | Linear | Class1 | 0.426 | 168.792 | 0.003 | 0.998 |
| M9[3333][3333] | depressive symptoms score | Quadratic | Class1 | -0.242 | 92.068 | -0.003 | 0.998 |
| M9[3333][3333] | depressive symptoms score | Cubic | Class1 | 0.043 | 15.345 | 0.003 | 0.998 |
| M9[3333][3333] | depressive symptoms score | Intercept | Class2 | 0.942 | 213.920 | 0.004 | 0.996 |
| M9[3333][3333] | depressive symptoms score | Linear | Class2 | 0.172 | 392.188 | 0.000 | 1.000 |
| M9[3333][3333] | depressive symptoms score | Quadratic | Class2 | -0.236 | 213.920 | -0.001 | 0.999 |
| M9[3333][3333] | depressive symptoms score | Cubic | Class2 | 0.064 | 35.653 | 0.002 | 0.999 |
| M9[3333][3333] | depressive symptoms score | Intercept | Class3 | 1.937 | 354.404 | 0.005 | 0.996 |
| M9[3333][3333] | depressive symptoms score | Linear | Class3 | 0.073 | 649.741 | 0.000 | 1.000 |
| M9[3333][3333] | depressive symptoms score | Quadratic | Class3 | -0.121 | 354.404 | -0.000 | 1.000 |
| M9[3333][3333] | depressive symptoms score | Cubic | Class3 | 0.028 | 59.067 | 0.000 | 1.000 |
| M9[3333][3333] | depressive symptoms score | Intercept | Class4 | 3.987 | 1248.258 | 0.003 | 0.997 |
| M9[3333][3333] | depressive symptoms score | Linear | Class4 | 0.608 | 2288.474 | 0.000 | 1.000 |
| M9[3333][3333] | depressive symptoms score | Quadratic | Class4 | 0.088 | 1248.258 | 0.000 | 1.000 |
| M9[3333][3333] | depressive symptoms score | Cubic | Class4 | -0.078 | 208.043 | -0.000 | 1.000 |

**Supplemental Tables S5**. The best trajectory model applicability.

| **GROUP** | **shape** | **avepp** | **COUNT** | **Pj** | **πj** | **occ_c** | **entropy_K** |
| --- | --- | --- | --- | --- | --- | --- | --- |
| Group1 | Linear | 88.911 | 1602 | 44.451 | 44.070 | 10.176 | 0.770 |
| Group2 | Linear | 82.362 | 1362 | 37.791 | 37.489 | 7.786 |  |
| Group3 | Linear | 84.831 | 413 | 11.459 | 12.114 | 40.573 |  |
| Group4 | Linear | 92.755 | 227 | 6.299 | 6.327 | 189.546 |  |
